# Differential diagnosis of lung cancer, its metastasis and chronic obstructive pulmonary disease based on serum Vegf, Il-8 and MMP-9

**DOI:** 10.1038/srep36065

**Published:** 2016-11-04

**Authors:** Murali M. S. Balla, Sejal Desai, Pallavi Purwar, Amit Kumar, Prashant Bhandarkar, Yogesh K. Shejul, C. S. Pramesh, S. Laskar, Badri N. Pandey

**Affiliations:** 1 Radiation Biology and Health Sciences Division, Bhabha Atomic Research Centre, Trombay, Mumbai 400 085, India; 2 Homi Bhabha National Institute, Anushakti Nagar, Mumbai 400 094, India; 3 Tata Memorial Hospital, Dr E. Borges Road, Parel, Mumbai, 400012, India; 4 Medical Division, Bhabha Atomic Research Centre, Anushakti Nagar, Mumbai 400 094, India.

## Abstract

Chronic obstructive pulmonary disease (COPD) patients are at higher risk of developing lung cancer and its metastasis, but no suitable biomarker has been reported for differential diagnosis of these patients. Levels of serum biomarkers (VEGF, IL-8, MMP-9 and MMP-2) were analyzed in these patients, which were compared with healthy donors (HD). Levels of VEGF (P < 0.005) and MMP-9 (P < 0.05) were significantly higher in COPD patients than HD. Compared to HD, a decrease in IL-8 (~8.1 folds; P < 0.0001) but an increase in MMP-9 (~1.6 folds; P < 0.05) levels were observed in the lung cancer patients. Cancer patients showed significantly (P < 0.005) lower levels of serum VEGF (1.9 folds) and IL-8 (~9 folds) than the COPD patients. VEGF level was significantly higher (2.6 folds; P < 0.0005) in metastatic than non-metastatic cancer patients. However, MMP-2 didn’t show significant variation in these patients. The Youden’s index (YI) values for lung cancer diagnosis in HD using IL-8 was 0.55 with 83.3% overall accuracy. VEGF was able to diagnose COPD in HD with better YI (0.38) and overall accuracy (70.6%). IL-8 was able to diagnose cancer in COPD patients and HD with YI values of 0.35, 0.55 with 71% and 83.3% overall accuracy, respectively.

According to World Health Organization Report (2012) chronic obstructive pulmonary disease (COPD) and lung cancer are the most rapidly growing diseases globally and fall within the top 10 causes of death. COPD is the third and lung cancer (with trachea and bronchus cancer) is the fourth worldwide health problem with 3.1 and 1.6 million annual deaths, respectively^1^. Interestingly, COPD and lung cancer are interlinked closely with cause and effect relationship^2^,^3^,^4^. Both the diseases are known to be induced by exposure of toxic gases and air pollutants especially cigarette/tobacco smoke, which initially induce systemic inflammatory changes and subsequently localized chronic inflammation in the lungs. About 8–50% prevalence of COPD is reported in patients diagnosed with lung cancer^5^, which are mostly associated with obstruction of air passage to lungs due to tumor mass. The association of these two diseases becomes further critical as COPD patients are reported to be at higher risk to develop lung cancer^6^,^7^. In a matched case-control study, it was found that ~1% of COPD patients develop lung cancer each year, while only 0.2% of patients with normal pulmonary function develop lung cancer (~5 folds increase in risk of lung cancer)^7^. According to a recent study, smoking poses almost four folds higher risk of developing lung cancer than non-smoking COPD patients^8^. It is interesting to note that the histological characteristics of lung cancer developed in COPD patients are different than those developed in non-COPD patients^9^. Management of lung cancer with COPD condition adds another line of complication as these patients show meager prognosis because of poor pulmonary function and quality of life in these patients^5^. Moreover, lung cancers developed in COPD patients tended to be at the higher grade of malignancy. The five year survival rate in lung cancer patients with COPD was significantly lower than patients with normal pulmonary function (77% versus 91.6%) due to higher recurrence rate^10^.

Usually lung cancer is asymptomatic until the disease is already at an advanced stage. Many times, lung cancer symptoms are mistaken with other problems, such as infection or effect of smoking, which further delays the diagnosis. Therefore, majority of lung cancer cases are diagnosed at either stage III or IV, thus making curative surgery difficult for the clinicians. In this direction, chest radiography and sputum cytology had been employed for lung cancer screening in the 1980 s. However, noticeable advantage in terms of decrease in patient mortality due to the screening remains inconclusive^11^. Even though, low dose computed tomography achieved early detection and increased resectability rates for lung cancer, its mortality benefits have not been proved^12^. Initial results from National Lung Screening Trail in USA showed 20% fewer lung cancer deaths among trail participants compared with subjects screened by chest radiography^13^. However, being radiation based and costly technique, routine use of computed tomography for mass screening of patients is rather impractical and unaffordable, especially in developing countries like India. In this direction, the potential of biomarkers (e.g. circulating DNA in peripheral blood, circulating tumor-derived exosome, micro-RNAs, comparative protein profiling) in lung cancer diagnosis has been discussed and reviewed^14^. Recently, evaluation of VEGF-C and other tumor markers in bronchoalveolar lavage fluid showed ability of neuron-specific enolase for lung cancer diagnosis^15^. However, serum cytokine based markers to differentially diagnose lung cancer, its metastasis and COPD patients is not known to the best of our knowledge. Compared to other techniques, serum cytokine analysis using ELISA could be better suited for lung cancer screening/diagnosis due to the ease in sample collection with quick and economic way to obtain the results.

Metastasis is a critical clinical factor in the disease management perspective of COPD. A large variety of inflammatory mediators present in the lungs of COPD patients promote the development of lung cancer and subsequent metastasis^16^,^17^. In this regard, developing suitable biomarkers for early diagnosis of lung cancer/its metastasis in COPD patients, may help for lung cancer surveillance and hence better management of the disease. In this regard, our previous study in human lung cancer cells (A549) showed role of autocrine cytokines [vascular endothelial growth factor (VEGF), interleukin-8 (IL-8), matrix metallo-protease-2 (MMP-2)] in the process of epithelial-to-mesenchymal transition and associated metastatic features^18^,^19^. This study is a further attempt to distinguish lung cancer (metastatic and non-metastatic) and COPD conditions by assaying serum levels of these analytes.

## Results

### Serum levels of VEGF, IL-8, MMP-9 and MMP-2 in COPD and lung cancer patients

**Figure 1:**
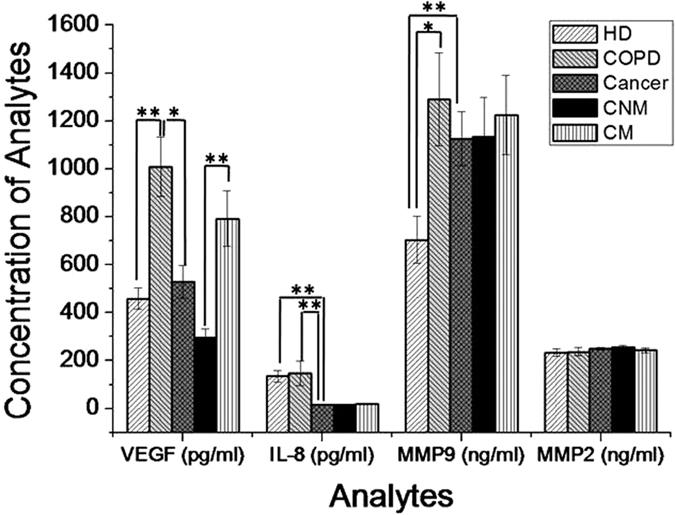
Levels of serum analytes in healthy donors, COPD and lung cancer patients. *Significantly different to each other at P < 0.05; **significantly different to each other at p < 0.005. HD: healthy donors; COPD: Chronic Obstructive Pulmonary Disease; Cancer: Lung Cancer; CNM: Cancer non-metastatic; and CM: Cancer metastatic.

**Figure 2:**
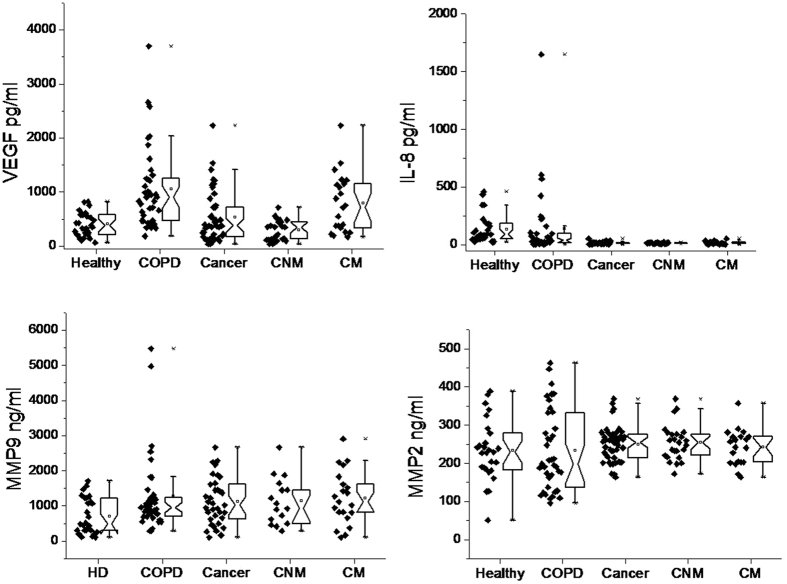
Individual values for different analytes were shown along with respective box-and-whisker plots. The notched point of plots show the mean value and upper and lower parts represent 25 and 75%, respectively. x: represent lower or upper values of the range. Abbreviations are same as Fig. 1.

Levels of VEGF, IL-8, matrix metallo-protease-9 (MMP-9) and MMP-2 were measured in serum samples of COPD and lung cancer patients, which were compared with HD (Figs 1 and 2, Table 1). Significantly higher (P < 0.0001) levels of serum VEGF (~2.1 folds; COPD: 1009.2 ± 122.8 pg/ml; HD: 459 ± 45.3 pg/ml) and MMP-9 (1.8 folds; COPD: 1289.6 ± 193.3 ng/ml; HD: 703.6 ± 98.2 ng/ml) were observed in COPD patients than HD. However, changes in the levels of IL-8 and MMP-2 were found to be insignificant between COPD patients and HD. Furthermore, results obtained in lung cancer patients were compared with other groups. Compared to HD, levels of VEGF and MMP-2 were insignificantly changed in lung cancer patients. Interestingly, level of IL-8 was found to be decreased by ~8.1 folds (cancer: 16.5 ± 1.3 pg/ml; HD: 134.4 ± 23.9 pg/ml; P < 0.0001) but MMP-9 level was found to be increased by ~1.6 folds (cancer: 1126.2 ± 112.4 ng/ml; HD: 703.6 ± 98.2 ng/ml; P < 0.05) in lung cancer patients compared to HD. Compared to COPD patients, insignificant change in the levels of MMP-9 and MMP-2 were observed in the serum samples of lung cancer patients. However, the levels of VEGF (1.9 folds; cancer: 528.3 ± 67.9 pg/ml; COPD: 1009.2 ± 122.8 pg/ml) and IL-8 (~9 folds; cancer: 16.5 ± 1.3 pg/ml; COPD: 146.7 ± 50.9 pg/ml) were significantly lower (P < 0.005) in lung cancer patients than COPD patients. Among the cancer patients, level of serum VEGF was significantly higher (2.6 folds, P < 0.0005) in metastatic (792.8 ± 115.7 pg/ml) than non-metastatic (295.6 ± 38.5 pg/ml) patients. Other serum biomarkers showed insignificant difference between non-metastatic and metastatic groups of patients. In the two COPD patients (out of 39), who were incidentally diagnosed with lung cancer metastasis, the levels of these serum biomarkers were determined [VEGF (2658.0, 1013.5 pg/ml), IL-8 (11.8, 23.7 pg/ml), MMP-9 (661.8, 1166.7 ng/ml) and MMP-2 (209.2, 95.7 ng/ml)]. In these patients, except for MMP-2, the levels of other biomarkers (VEGF, IL-8 and MMP-9) followed a close proximity to COPD and lung cancer patients, but clearly differed from HD.

### Diagnostic ability of serum biomarkers for lung cancer, its metastasis and COPD

**Figure 3:**
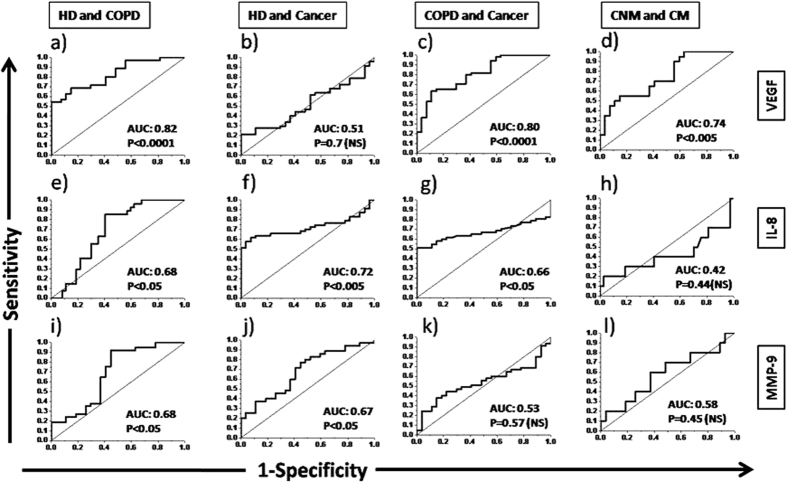
ROC curves of different analytes between various groups. Abbreviations are same as Fig. 1. NS: Non-significant.

The area under curve (AUC) values for VEGF to diagnose COPD in HD and cancer in COPD patients were found to be 0.82 and 0.80, respectively (Fig. 3). Serum VEGF has also shown good sensitivity to distinguish between metastatic and non-metastatic lung cancer patients (AUC: 0.74). Serum IL-8 showed good sensitivity to diagnose cancer in HD (AUC: 0.72) and cancer in COPD patients (AUC: 0.66). But, IL-8 didn’t show good sensitivity to distinguish between metastatic and non-metastatic lung cancer patients. MMP-9 showed its ability to fairly diagnose COPD (AUC: 0.68) and cancer (AUC: 0.67) in healthy subjects. Other patient groups showed lower AUC values (<0.6) for other analytes suggesting their poor diagnostic ability.

The upper or lower threshold values (mean of 95% CI) for the analytes were determined in different patient groups (COPD and cancer) depending on their increase or decrease in serum compared to HD. On this basis, the upper CI of mean for VEGF and MMP-9 were found to be >552.8 pg/ml and >905.6 ng/ml, respectively, however, the lower CI of mean for IL-8 was <85.1 pg/ml. Based on these cut off values, the diagnostic ability of these analytes were evaluated for calculating the Youden’s index (YI) and the overall accuracy of the test. These values were calculated for VEGF, IL-8 and MMP-9 between four groups i.e. (i) HD and COPD, (ii) HD and cancer, (iii) COPD and cancer and (iv) non-metastatic and metastatic (Table 2). For VEGF, YI values were found to be 0.38 and 0.33 with 70.6% and 65% overall accuracy for diagnosis of COPD in HD and cancer in COPD patients, respectively. However, IL-8 was able to diagnose cancer in COPD patients and HD with YI 0.35, 0.55 and 71%, 83.3% overall accuracy, respectively. For diagnosis of COPD in HD, serum MMP-9 showed YI of 0.21 with overall accuracy 60.3%. Serum MMP-9 showed almost similar ability to diagnose cancer in healthy subjects (YI: 0.17; overall accuracy: 58.1%). The YI and overall accuracy values for VEGF, IL-8 and MMP-9 were found to be very low with poor diagnostic ability for other combination of HD/patients.

## Discussion

Previously, our *in-vitro* study has shown that cytokines (VEGF, IL-8 and MMP-2) secreted from A549 cells result in metastatic features in these cancer cells^18^,^19^. To further evaluate the changes of these biomarkers in clinical conditions, levels of these serum markers were monitored in non-metastatic and metastatic lung cancer patients. These results were compared with COPD patients, who are at high risk of developing lung cancer. The important characteristics of COPD is the increased recruitment of inflammatory cells (such as CD8^+^ T-lymphocytes, neutrophils and macrophages) producing pro-inflammatory cytokines (like VEGF, TNF-α, IL-1, IL-6 and IL-8, IL-17) in the lung tissue^20^. Moreover, vascular remodeling occurs during COPD pathogenesis^21^, which involves angiogenesis, bronchial vascularization and structural changes in vascular walls. The increased level of serum VEGF in COPD patients may be associated with these inflammatory and angiogenic changes. Moreover, the level of VEGF has been reported to be differentially modulated depending on the lung condition. In case of chronic bronchitis, VEGF level was found to be increased, however, in case of emphysema it was found to be decreased^22^ with lower expression of VEGF receptors^23^. The lower serum level of VEGF in lung cancer patients (than the COPD) could be due to its utilization by proliferating tumor cells and its vasculature, which needs further investigation. In order to metastasize in the distant organs, tumor cells need to degrade basement membrane and gain entry into the systemic circulation through tumor vasculature^24^,^25^,^26^. VEGF secreted by tumor and/or other cells of tumor microenvironment is the key mediator of vasculogenesis, which plays a crucial role in the process of metastasis. These facts support our observation of significantly increased level of VEGF in metastatic than non-metastatic lung cancer patients. MMP-9 has been known to cause emphysema in COPD and angiogenesis/metastasis during lung cancer^16^, which supports our observation of increased serum levels of MMP-9 in COPD and lung cancer patients. However, we didn’t find significant difference between the levels of serum MMP-9 in metastatic and non-metastatic lung cancer patients. These results are in agreement with recent meta-analysis, where the activity but the not the level of MMP-9 in serum was found to be correlated with lung cancer metastasis^27^. Moreover, our results also showed increased levels of serum VEGF and MMP-9 in lung cancer patients compared to the healthy donors, which are in agreement with studies showing higher level of these markers in lung cancer patients compared to benign or normal counterparts^28^,^29^. Even though, majority of literature suggest increased level of IL-8 in lung cancer patients^30^,^31^,^32^, our results showed decreased IL-8 in serum samples of cancer patients than HD/COPD patients. Our results get supported by study in which human lung cancer cells (A549) clones (3B4) producing low level of IL-8 showed higher proliferation than clones (2B2) producing higher level of IL-8^33^. It is possible that the higher proliferation rate in lung cancer may be associated with the lower level of IL-8 as observed in our study. Our results were also supported by decreased level of serum IL-8 in the COPD patients, who were incidentally diagnosed with lung cancer. Compared to HD, the level of serum MMP-9 was significantly (P < 0.05) increased in case of COPD and lung cancer patients. However, it did not show difference between COPD and lung cancer patients. MMP-2 didn’t show any difference amongst HD, COPD and lung cancer patients, which is not in agreement in change in the level of serum MMP-2 reported in lung cancer patients^34^,^35^. In addition, MMP-2 was found to be better prognostic marker in non-small cell lung carcinoma patients than MMP-9^36^. However, our results are in agreement of another study, which showed MMP-9 as better prognostic marker than MMP-2 in patients with mixed types of lung cancer^37^. A limited literature exists studying the level of MMP-2 in COPD patients, which however were contradictory in terms of increased^38^ or decreased^39^ levels in COPD patients compared to healthy subjects. Our results also showed slight decrease in the level of MMP-2 in COPD patients (than HD), which is in agreement with the recent study^39^. Moreover, this study also showed genetic polymorphism and protein level variation in COPD patients of Mexican population. Even though, such genetic and protein level variation for MMP-2 is not known in the Indian population, its existence may cause higher variability of the biomarker and hence, non-significant changes between various patient groups as observed in our study.

The changes in level of these serum biomarkers were further analysed for their diagnostic ability by plotting the ROC curves (Fig. 3a–c) and calculation of Youden’s Index (Table 2). Ability of MMP-9 and VEGF to diagnose COPD patients in HD was found better (YI: 0.21 and 0.38, AUC: 0.68 and 0.82) than IL-8. Moreover, the ability of serum MMP-9 (AUC: 0.67; P < 0.05) and IL-8 (AUC: 0.72; P < 0.005) to diagnose cancer (from HD) were found to be significant. On the other hand, decrease in IL-8 (YI: 0.35; AUC: 0.66; P < 0.05) and increase in VEGF (YI: 0.33; AUC: 0.80; P < 0.0001) showed significant ability to diagnose cancer in COPD patients. For differential diagnosis between non-metastatic and metastatic patients, VEGF showed better AUC (0.74; P < 0.005) value than other markers. The lower YI (0.28) in this condition seems to be associated with lower sample size. Based on 95% CI of mean, HD can be differentially diagnosed as COPD with serum levels of VEGF (801.9–1306.5 pg/ml) and MMP-9 (907.4–1649.3 ng/ml). However, COPD patients can be diagnosed as lung cancer with serum level of IL-8 (13.9–19.2 pg/ml). Within lung cancer patients, VEGF (552.1–1033.5 pg/ml) can be used to differentially diagnose the metastatic condition. It may also be important to emphasize here that the overall accuracy was found to be high (65–80%) for the serum biomarkers suggesting their significant differential diagnostic ability for lung cancer, its metastasis and COPD. However, further studies are required to evaluate the variation of these biomarkers in COPD patients, who are at high-risk to develop lung cancer. Moreover, a follow up in non-metastatic patients, which may eventually become metastatic, would help to decide the threshold values for these biomarkers for better management of lung cancer patients.

## Materials and Methods

### Patients and serum collection

COPD patients (n = 39) were from BARC Hospital, Mumbai. Out of them two patients were later diagnosed with lung cancer metastasis and were excluded from the analysis. Serum samples of lung cancer patients (n = 45) were obtained from the depository (stored at liquid nitrogen) at Tata Memorial Hospital, Mumbai. Out of 45 lung cancer patients, 20 patients were diagnosed to be metastatic. Wherever required, analysis of lung cancer patient’s samples was performed either as all lung cancer samples together (cancer) or as non-metastatic (CNM) and metastatic (CM) patient groups. Serum samples from age matched healthy donors (n = 27) were collected from the volunteers, who came for regular checkup at Bhabha Atomic Research Centre Hospital, Mumbai. These patients were non-COPD and without past history of any lung disorder. Details of HD, COPD and lung cancer patients are provided in Table 3. The methods were carried out in accordance with the approved experimental protocols and guidelines of Institutional Ethical Committees at BARC Hospital, Mumbai (HD and COPD patients) and Tata Memorial Hospital, Mumbai (lung cancer patients). Written informed consent was obtained from all the subjects for the collection of samples. Fresh serum samples were isolated from 5 ml blood obtained from COPD and HD, which was stored at −80 °C until use.

### Enzyme Linked Immunosorbent Assay (ELISA) for VEGF, IL-8, MMP-9 and MMP-2 in serum samples

VEGF, IL-8, MMP-9 and MMP-2 levels in serum samples were quantified using ELISA kits (Quantikine ELISA, R&D Systems, USA) following standard procedure provided along with the kits. For MMP-9, 35 lung cancer serum samples (CNM: 16; CM: 19) were analysed. For other analytes, sample numbers remain the same as mentioned in the previous section (Table 3). Serum samples stored at −80° were thawed to room temperature and where ever required dilutions were made (1:20 for MMP-2 and 1:100 for MMP-9). Assay buffer (100 μl) and serum samples (100 μl for VEGF and MMP-9 and 50 μl for MMP-2 and IL-8) were added to the wells of a micro-titer plate coated with respective human specific VEGF, IL-8, MMP-9 and MMP-2 antibody followed by incubation (2 h; room temperature) on a horizontal orbital micro plate shaker. After incubation, plates were washed with buffer three times for 5 min each. Then 200 μl of respective conjugates (VEGF, IL-8, MMP-9 and MMP-2) were added to respective plates, and then incubated for 2 h at room temperature. Further, 200 μl of substrate solution was added to each well and incubated for 30 min at room temperature in the dark. Finally, after the addition of 50 μl of stop solution, color development was determined at 450 and 540 nm using micro plate reader (Infinite, M-200 PRO, Tecan, Switzerland). Absorbance at 540 nm was subtracted from the reading at 450 nm and analyte concentrations were calculated from the respective standard curves.

### Statistical Analysis

Statistical analysis was done using OriginPro 8.0 software. Frequency distribution values of analytes were represented in mean ± SEM. The significance between different groups was evaluated by un-paired t test. To evaluate the diagnostic ability of these analytes, AUC values were determined from receiver operating characteristics (ROC) curves [sensitivity versus (1 − specificity)] for the selected patient groups/analytes. This kind of calculation was made as our data has more number of discrete values on continuous rating scale. AUC curves were prepared for each of the three analytes (i.e. VEGF, IL-8 and MMP-9) between (i) HD and COPD (to diagnose COPD in HD), (ii) HD and cancer (to diagnose cancer in HD), (iii) COPD and cancer (to diagnose cancer in COPD patients) and (iv) non-metastatic and metastatic (to distinguish between metastatic and non-metastatic lung cancer patients). Youden’s index was calculated by determining cut off values either by taking upper or lower 95% CI of mean (depending on the upward/downward trend of the particular analyte) compared to healthy donors. Sensitivity and specificity was calculated based on the number of cases that meet the conditions of lower or higher than the cut off values. Overall accuracy for diagnostic test was calculated as following: (sum of true negatives and true positives)/(sum of true negatives, true positives, false negatives and false positives) × 100.

## Additional Information

**How to cite this article**: Balla, M. M. S. *et al*. Differential diagnosis of lung cancer, its metastasis and chronic obstructive pulmonary disease based on serum Vegf, Il-8 and MMP-9. *Sci. Rep*. **6**, 36065; doi: 10.1038/srep36065 (2016).

**Publisher’s note:** Springer Nature remains neutral with regard to jurisdictional claims in published maps and institutional affiliations.

## Figures and Tables

**Table 1:** Serum levels of analytes in various groups.

Groups	Mean ± SEM Values of Analytes (95% CI of mean)			
	VEGF (pg/ml)	IL-8 (pg/ml)	MMP-9 (ng/ml)	MMP-2 (ng/ml)
HD	459 ± 45.3(366.3–552.8)	134.4 ± 23.9(85.1–183.7)	703.6 ± 98.2(501.6–905.6)	233.2 ± 15.2(201.9–264.4)
COPD	1009.2 ± 122.8(801.9–1306.5)	146.7 ± 50.9(41.6–237.9)	1289.6 ± 193.3(907.4–1649.3)	237.2 ± 17.2(198.9–266.8)
Cancer	528.3 ± 67.9(391.6–665)	16.5 ± 1.3(13.9–19.2)	1126.2 ± 112.4(897.8–1354.7)	249.4 ± 6.9(235.4–263.4)
CNM	295.6 ± 38.5(216.1–375.1)	15.5 ± 0.8(13.7–17.2)	1132.7 ± 166(778.9–1486.5)	254.6 ± 9.3(235.2–274)
CM	792.8 ± 115.7(552.1–1033.5)	17.8 ± 2.6(12.2–23.3)	1225.0 ± 165.8(879–1571.1)	242.9 ± 10.4(221.1–264.7)

**Table 2:** Youden’s index to diagnose COPD and cancer from healthy donors.

Analyte at 95% CI of mean	HD versus COPD	HD versus Cancer	COPD versus Cancer	CNM versus CM
VEGF >552.8 pg/ml (upper limit)	0.38 (70.6%)	0.10 (59.7%)	0.33 (65%)	0.28 (63%)
IL-8 <85.1 pg/ml (lower limit)	0.28 (66.2%)	0.55 (83.3%)	0.35 (71%)	0
MMP-9 >905.6 ng/ml (upper limit)	0.21 (60.3%)	0.17 (58.1%)	0.05 (51%)	0.03 (51.4%)

Youden’s index (scale of 0–1) was calculated as mentioned in materials and methods. The values in parentheses represent overall accuracy.

**Table 3:** Details of healthy donors, and COPD and lung cancer patients.

Parameters	Healthy Donors	COPD	Lung cancer
Number	27	37	45
Age (mean ± SD)	61.1 ± 10.4	70.1 ± 10.2	56.7 ± 10.6
Gender			
Male	20	19	41
Female	7	18	4
Lung Cancer Patients Categories			
Non-Metastatic		25	
Adenocarcinoma	—	—	22
Squamous cell carcinoma	—	—	3
Metastatic			20
Adenocarcinoma	—	—	15
Squamous cell carcinoma	—	—	5
